# A Dataset of physical-layer measurements in indoor wireless jamming scenarios

**DOI:** 10.1016/j.dib.2022.108773

**Published:** 2022-11-23

**Authors:** Saeif Alhazbi, Savio Sciancalepore, Gabriele Oligeri

**Affiliations:** a Division of Information and Computing Technology, College of Science and Engineering, Hamad Bin Khalifa University, Doha, Qatar; b Eindhoven University of Technology (TU/e), Eindhoven, Netherlands

**Keywords:** Software defined radios, IQ samples, BPSK, USRP X310

## Abstract

The broadcast nature of wireless communications makes them vulnerable to denial-of-service attacks. Indeed, an adversary can prevent the reception of wireless messages by transmitting signals with high power over the same frequency of the considered channel. This paper presents an experimental dataset of real-world indoor communication scenarios affected by different jamming techniques. Specifically, our dataset includes data acquired from 7 different Software Defined Radios (SDRs), i.e., the USRP Ettus Research X310, operating in an office environment. Each experiment is characterized by a transmitter, a receiver, and a jammer. While the hardware of the transmitter and the receiver are kept the same for all the experiments, the hardware of the jammer is changed adopting 5 different radios of the same brand. The dataset includes different jamming behaviors, based on the type of signal injected by the jammer: no jamming (silent), tone (sinusoidal), and Gaussian noise. Moreover, besides having multiple jamming devices and modes, the dataset also includes different transmission distances and jamming powers. In each experiment, a pre-determined sequence of bits has been modulated using the BPSK scheme, transmitted wirelessly under different jamming conditions, and then stored, at the receiver, as a 2-columns matrix of I/Q samples. Researchers can use this dataset in several ways, including: (i) developing active and reactive techniques for jamming detection, (ii) jamming identification at the physical layer, and finally, (iii) developing mitigation techniques supported by real data.

**Specifications Table**

**Table:** 

Subject	Computer Science: Computer Networks and Communications
Specific subject area	Physical-layer wireless security
Type of data	Table
How the data were acquired	We acquired the data by conducting real measurements in an indoor environment, using seven (7) different SDRs (USRP Ettus Research X310), each equipped with a daughterboard UBX160 and an Omni-directional VERT2450 stylo antenna. To implement the signal processing modules, we connected the SDRs via Ethernet to two Dell XPS15 9560 laptops, running GNU Radio version 3.8.
Data format	A list of files with .MAT extension (standard MATLAB format).
Description of data collection	The dataset contains physical-layer data (I-Q samples), gathered in various conditions including multiple jamming devices, various distances, and different jamming power levels. Overall, the dataset consists of 31 .MAT files, each one including 3 matrices representing the jamming mode, i.e., Tone jammer (Sine), Gaussian, and No jamming (Silent) in a specific measurement condition. Each matrix has two columns: the first represents the I component of the received signal while the second one represents the Q component.
Data source location	Institution: Hamad Bin Khalifa University City/Town/Region: Doha Country: Qatar Latitude and longitude (and GPS coordinates, if possible) for collected samples/data: [25.31557, 51.43446]
Data accessibility	Repository name: Zenodo Data identification number: 10.5281/zenodo.7119040 Direct URL to data: https://zenodo.org/record/7119040#.YzvJcnZBwmA
Related research article	Saeif AlHazbi, Savio Sciancalepore, Gabriele Oligeri. *BloodHound: Early Detection and Identification of Jamming at the PHY-layer*. To appear in IEEE Consumer Communications & Networking Conference (CCNC2023), 8-11 January 2023, Las Vegas, NV, US.

**Value of the Data**

• The acquired I/Q samples enable the research community to study jamming scenarios and develop methods for the detection and identification of jamming at the physical-layer.
• Researchers may test new jamming detection techniques using the proposed dataset.
• Researchers can evaluate jamming detection capabilities in a wide range of jamming scenarios, including several jamming hardware devices, different transmission distances and jamming power levels.

## 1 Objective

Physical-layer information from wireless communications under a variety of jamming scenarios allow the development of jamming detection and identification techniques. Furthermore, since there is a scarcity of experimental jamming datasets generated by real physical devices, we wanted to provide the research community with such an information, to push further the research on jamming detection, identification, and response.

## 2 Data Description

Table 1 describes the dataset structure as well as the experimental settings adopted to generate the data. The dataset is constituted by 31 MAT files, each one containing three matrices representing the jamming modes: “Nojamming”, “Sine”, and “Gaussian”. Each matrix is constituted by two-columns, representing the I (first column) and the Q samples (second column), respectively. In each experiment, the devices with ID 2 and ID 3 act as transmitter and receiver, respectively, while the remaining devices, having IDs from 4 to 8, act exclusively as jammers.

**Table 1:** Files included in the dataset along with the specific experiment parameters.

File Name	Transmitter ID	Receiver ID	Jammer ID	Relative Jamming Power	Distance between Tx and Rx [meters]	Measurement duration [Seconds]
W1	2	3	4	0.1	10	600
W2	2	3	4	0.3	10	600
W3	2	3	4	0.6	10	600
W4	2	3	5	0.1	10	600
W5	2	3	5	0.3	10	600
W6	2	3	5	0.6	10	600
W7	2	3	4	0.2	10	600
W8	2	3	5	0.2	10	600
W9	2	3	4	0.4	10	600
W10	2	3	5	0.4	10	600
W11	2	3	5	0.5	10	600
W12	2	3	4	0.5	10	600
W13	2	3	4	0.7	10	600
W14	2	3	5	0.7	10	600
W15	2	3	4	0.8	10	600
W16	2	3	5	0.8	10	600
W17	2	3	5	0.6	10	600
W18	2	3	4	0.6	10	600
W19	2	3	4	0.5	10	600
W20	2	3	5	0.5	10	600
W21	2	3	6	0.5	10	600
W22	2	3	7	0.5	10	600
W23	2	3	8	0.5	10	600
W24	2	3	4	0.5	3	600
W25	2	3	4	0.5	5	600
W26	2	3	4	0.5	7	600
W27	2	3	4	0.5	10	600
W28	2	3	4	0.5	13	600
W29	2	3	4	0.5	16	600
W30	2	3	4	0.5	19	600
W31	2	3	4	0.5	21	600

We considered three parameters: (i) the jamming device, (ii) the intensity of the jamming signal, and finally, (iii) the distance between the transmitter (TX) and the receiver (RX). We would like to stress that each file in the dataset contains 3 measures, i.e., two with jamming and one with no jamming, that might be used to characterize the radio channel in the absence of the jamming phenomena. More details on such a channel characterization can be found also in the paper obtained from this dataset [1].

**Varying the jamming power.** Measurements from W1 to W18 refer to the scenario where we fixed the distance between the transmitter and the receiver (10 m), while we considered 2 different jammers (ID 4 and 5), and we varied the relative jamming power between 0.1 and 0.8.

**Varying the jamming device.** Measurements from W19 to W23 refer to the scenario where the relative jamming power (0.5), distance (10 m), transmitter (ID = 2) and receiver (ID = 3) are fixed, while we considered different jamming radios (ID spanning from 4 to 8).

**Varying the distance between TX and RX.** Measurements from W24 to W31 refer to the scenario where we fixed the normalized jamming power to 0.5, the transmitter, the receiver and the jammer ID to the radios with ID 2, 3, and 4, respectively, and we changed the distance between the TX and the RX considering 3, 5, 7, 10, 13, 16, 19 and 21 meters.

To convert the normalized power values of the USRP x310 between decibel-milliwatts (dBm) and milliwatts (mW), we used an RF meter. The mapping is reported in Table 2.

**Table 2:** Mapping between Relative Power, dBm, and mW for the USRPX310.

Relative Power	dBm	mW
0.1	-13	0.05
0.2	-10	0.1
0.3	-7	0.2
0.4	-3.5	0.45
0.5	0	1
0.6	2.8	1.9
0.7	6	3.98
0.8	9	7.94
0.9	12	15.84
1	15	31.62

**Fig. 1:**
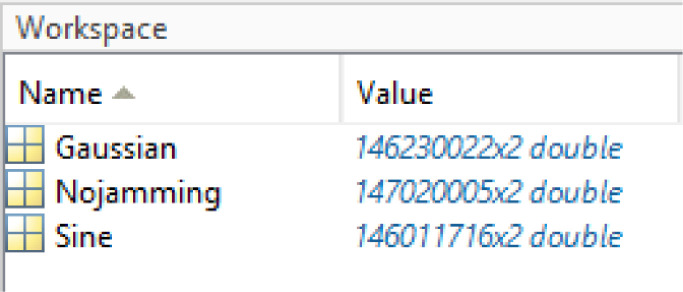
Snapshot of “w1.mat” content in MATLAB workspace.

The files can be imported into any tools that can process .MAT files, such as MATLAB. To import the data into MATLAB, the command ``load'' should be used. For example, when executing the command load with ‘w1.mat’, three matrices of type double and shape Nx2 are loaded to the MATLAB workspace, as depicted in Fig. 1.

*>> load('w1.mat')*

The names of the variables reported in Fig. 1 represent the previously mentioned jamming scenarios:

- Nojamming (Silent): Jammer is not transmitting
- Gaussian: Jammer transmitting a signal with Gaussian noise distribution.
- Sine: (Tone)-Jammer transmitting a sinusoidal signal.

**Fig. 2:**
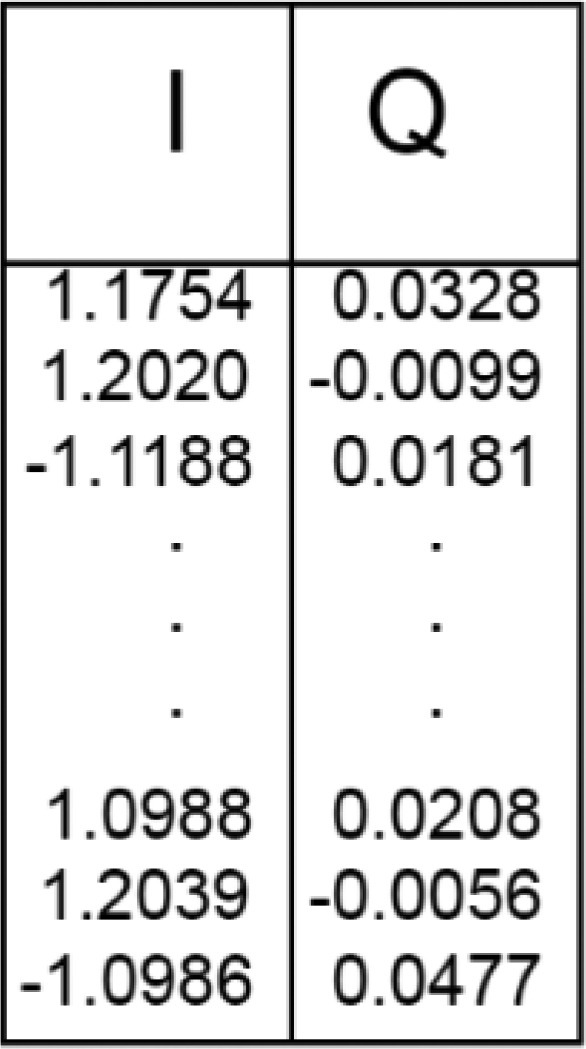
Content and shape of the two-columns matrix associated with the I-Q samples.

Note that the No-jamming measurements included in each file are different each other's; indeed, they have been executed right before the collection of the ``Sine'' and ``Gaussian'' measurements reported in the same file. We provide an example of the matrix containing the I-Q samples in Fig. 2. The first column corresponds to the I component of the complex signal, while the second column corresponds to the Q component.

## 3 Experimental Design, Materials and Methods

The analysis of the data described in this paper is presented in [1], while in the following we focus on the hardware and software set-up.

### 3.1 Hardware setup

**Fig. 3:**
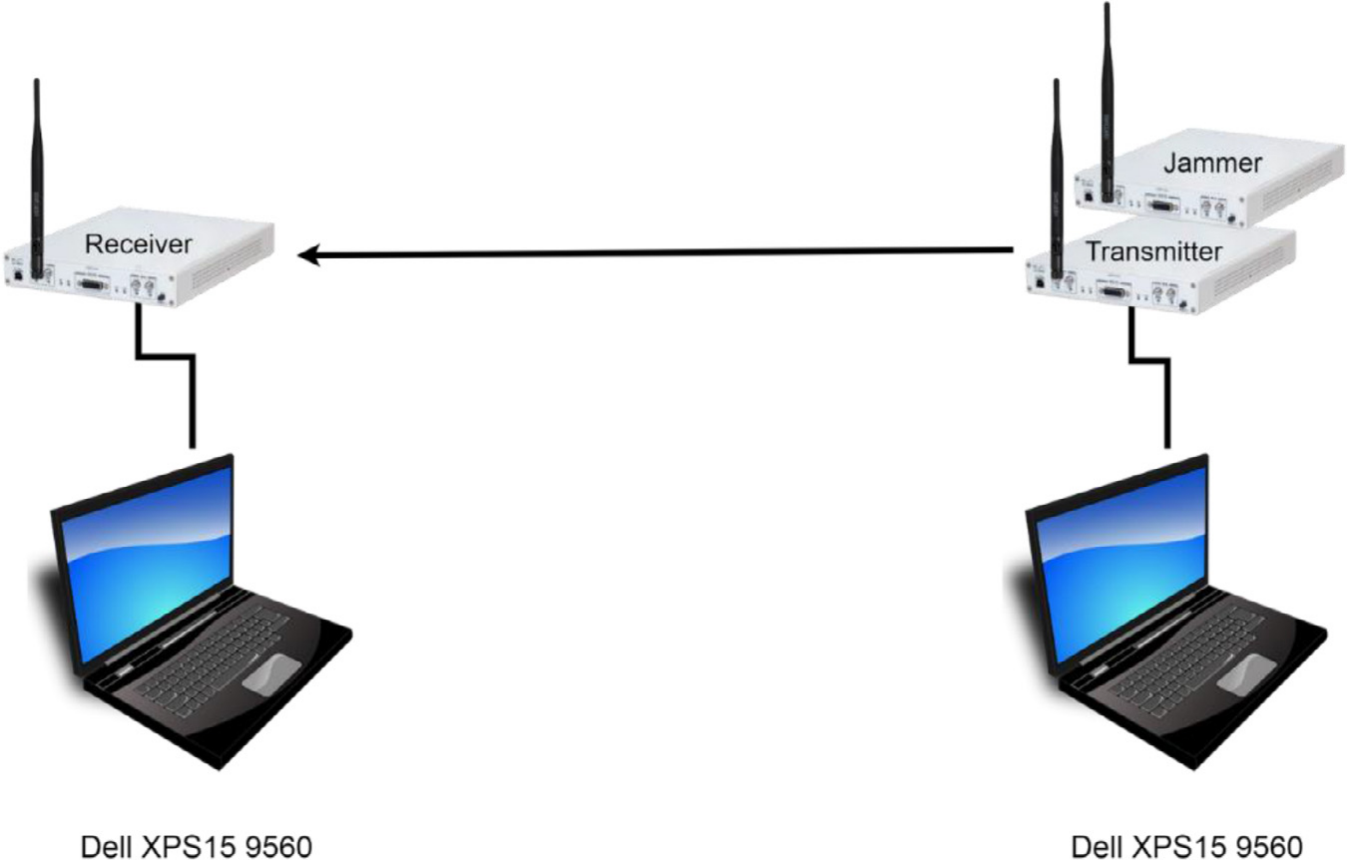
Hardware Setup for data collection.

Our measurement setup [2,3] includes 7 (seven) Ettus Research X310 [2] Software Defined Radios (SDRs), each equipped with a UBX160 daughterboard [3] and a VERT2450 Omni-directional stylo antenna from the same vendor. Fig. 3 shows the hardware setup adopted for data generation and collection. For all the measurements, the devices with ID 2 and 3 took the role of the transmitter and the receiver, respectively, while the remaining devices, with IDs 4 to 8, served as the jammer. We connected each of the SDR via an Ethernet cable to a Dell XPS15 9560 laptop equipped with 32GB RAM and a processor Intel core i5-7300HQ. The laptops run the GNU Radio development toolkit, one to control both data transmission and jamming, and the other one to handle data reception (see Software setup below).

**Fig. 4:**
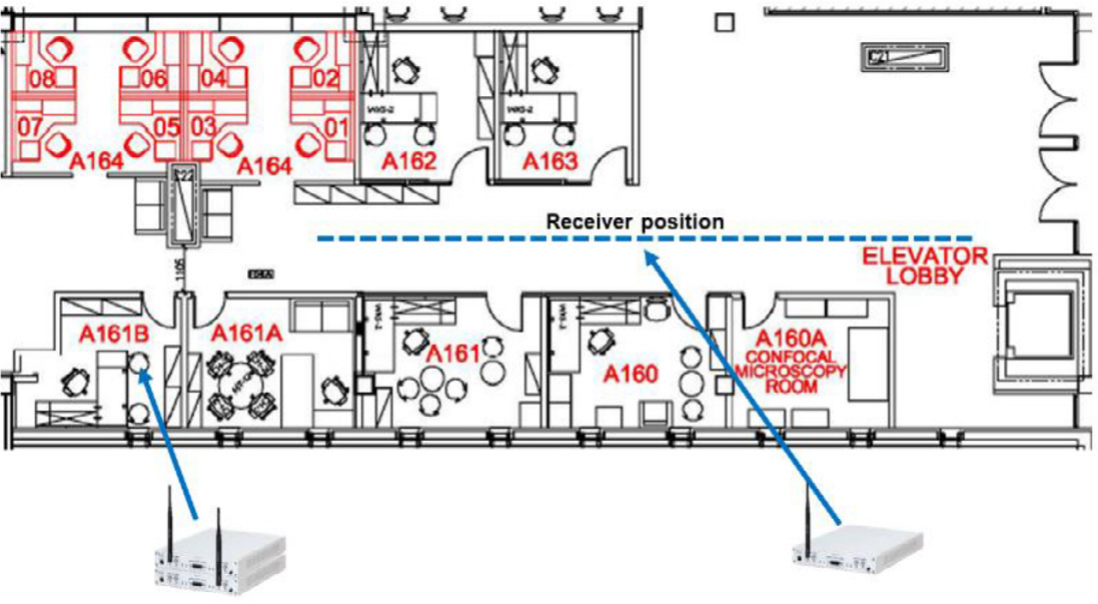
Measurement scenario: an office environment characterized by people moving around and non-Line-of-Sight.

During the different experiments, the position of the transmitter and the jammer was static, i.e., one on top of the other, while the receiver's location varied depending on the specific measurement conditions of interest, as depicted in Fig. 4. It is worth noting that all measurements were performed during working hours in an indoor office environment and with people moving in the proximity of the devices. Therefore, the experiments experienced dynamic channel conditions characterized by non-line-of-sight and multipath.

### 3.2 Software set-up

We adopted the GNU Radio Development Toolkit [4] (version 3.8) to control the SDRs. GNU Radio is a free and open-source development toolkit to design and implement applications running on SDRs, using signal processing blocks. For all the acquired measurements, we set the center frequency to 900 MHz, the sample rate to 1 million samples per second at both the transmitter's and receiver's side. The sample rate of the jammer has been set to 1Msps, as well. Furthermore, at the transmitter side, we used the constellation modulation block to encode and send a sequence of 256 bytes [0,…,255] using the Binary Phase Shift Keying (BPSK) modulation. We adjusted the transmission power and the receiver gain to 1, which corresponds to approximately 15 dBm (32mW) at the transmitter side. The GnuRadio blocks adopted by the receiver, the transmitter, and the jammer are described in the following:

Receiver:

• An*Adaptive Gain Control (AGC)* block, to control the amplitude of the received signal in the expected range of the BPSK modulation, mitigating any fluctuation caused by multipath fading.
• *A symbol sync* block, to perform time synchronization.
• A *Costas Loop* block, to recover the center frequency of the carrier and down-convert the signal to baseband.

It is worth noting that no channel estimation block or technique was used on the receiver side.

Transmitter:

• *File source* block, to read a file from the disk.
• *Constellation modulator* block, to modulate the bits sequence according to the BPSK modulation.
• *USRP sink* block, to transmit the signal over the radio spectrum.

Jammer:

• *NoiseSignal Source*, to generate two different waveforms (Sine jammer) or a digital gaussian distributed sequence (Gaussian jammer)
• *USRP Sink*, to transmit the jamming signal to the USRP device over the radio spectrum.

The GNU Radio block diagram for the jammer includes the following blocks:

• *Signal Source*, to generate two different waveforms (Sine jammer) or a digital gaussian distributed sequence (Gaussian jammer)
• *USRP Sink*, to transmit the signal to the USRP device.

## Ethics Statements

The current work does not involve human subjects, animal experiments, or any data collected from social media platforms.

## CRediT Author Statement

**Saeif Alhazbi:** Conceptualization, Methodology, Software, Validation, Investigation, Resources, Data Curation, Writing – original draft; **Gabriele Oligeri:** Conceptualization, Methodology, Software, Validation, Investigation, Resources, Data Curation, Writing – original draft; **Savio Sciancalepore:** Conceptualization, Methodology, Software, Validation, Investigation, Resources, Data Curation, Writing – original draft.

## Declaration of Competing Interest

The authors declare that they have no known competing financial interests or personal relationships that could have appeared to influence the work reported in this paper.

## Data Availability

A Dataset of Physical-Layer Measurements in Indoor Wireless Jamming Scenarios (Original data) (Zenodo).
